# Trends and hotspots in global influenza and intestinal flora research based on bibliometrics

**DOI:** 10.3389/fmicb.2025.1630924

**Published:** 2025-07-28

**Authors:** Dongsheng Wu, Tongtong Wang, Haoran Wu, Yuang Dong, Ziqin Huang, Jun Zhang, Wei Zhang

**Affiliations:** ^1^First Clinical Medical College of Anhui University of Traditional Chinese Medicine, Hefei, China; ^2^The First Affiliated Hospital of Hunan University of Traditional Chinese Medicine, Changsha, China; ^3^Shuguang Hospital, Affiliated with Shanghai University of Traditional Chinese Medicine, Shanghai, China

**Keywords:** influenza, intestinal flora, intestinal-lung axis immunomodulation, bibliometric analysis, research trends

## Abstract

**Objective:**

Influenza (hereinafter referred to as influenza) is a pandemic and seasonal respiratory infectious disease that can lead to a global pandemic, posing a major threat to global public health. Studies have shown that influenza can lead to an imbalance in the intestinal flora, and disruption of the intestinal flora can exacerbate the progression of the disease, suggesting a potential link between influenza and intestinal flora. There is still a lack of systematic summary of bibliometric analysis in this field, therefore, this study aims to reveal the research dynamics, collaborative networks and cutting-edge hotspots in the field of influenza-intestinal flora association through bibliometric methods.

**Methods:**

Bibliometric analysis was used to retrieve 554 papers on influenza and intestinal flora from the Web of Science Core Collection (WoSCC) database from 2011 to 2025. After screening, 283 papers were included, and co-occurrence and clustering analyses of countries, authors, institutions, journals, references, and keywords were performed using VOSviewer, CiteSpace, and Bibliometrix; statistical visualization was performed via Microsoft Excel.

**Results:**

China is the country with the highest number of published papers and the leading CSI in terms of international collaboration intensity. The most popular journal in this field is Frontiers in Microbiology with 20 publications, while the most influential journal is Nature with 605 citations. Zhejiang University was the institution with the highest number of publications and Francois Trottein was the most prolific author. Keyword co-occurrence analysis showed that gut microbiota, influenza, probiotics, intestinal microbiota and COVID-19 were the core research hotspots, and clustering analysis further revealed the “intestinal-pulmonary axis of immunoregulation,” such as Cluster analysis further revealed the “intestinal-lung axis immunoregulation,” such as Th17/Treg balance, short-chain fatty acids and probiotics, as the cutting edge.

**Conclusion:**

This study is the first to systematically map the bibliometrics of influenza and gut flora. The most influential countries, research institutions and researchers were identified through bibliometric analysis, showing the current research trends and hotspots in influenza and intestinal flora control. The results can provide theoretical guidance for future influenza prevention and control strategies targeting flora.

## 1 Introduction

Influenza is an acute respiratory infectious disease caused by influenza viruses, characterized by rapid transmission, frequent mutation, and widespread impact, which seriously threatens human health. Epidemiological studies have shown that influenza is closely associated with the onset of pneumonia, and the high mortality rate of hospitalized patients with influenza-associated pneumonia significantly increases the global public health burden (Koehler et al., 2019; Chow et al., 2019). Despite the role of vaccination and antiviral drugs in mitigating the consequences of influenza, rapid viral mutation, fluctuating vaccine efficacy, and antiviral drug resistance remain major challenges to current prevention and control efforts (Ni et al., 2019; JAMA, 2022). In recent years, there has been increasing evidence that the gut microbiota (GM) plays a crucial role in regulating immune function and participating in defense against viral infections (Kuiken et al., 2023; Harper et al., 2021). The gut microbiota, which consists mainly of bacteria, fungi, and viruses numbering more than 100 trillion, is a key player in maintaining host immune homeostasis (Altieri et al., 2023). Although the gut and lungs are not directly linked anatomically, they are linked through the “gut-lung axis” functional pathway. Disruption of the gut microbiota not only induces gut immune dysfunction, but may also affect lung antiviral immune responses through mechanisms such as systemic inflammation, metabolites, and immune regulation (Zhang et al., 2022; Pellegrini et al., 2020). For example, probiotic supplementation enhances host resistance to influenza viruses, whereas antibiotic-induced microbiota disruption weakens the immune efficacy of influenza vaccines (Stefan et al., 2020; Bordon, 2019). More importantly, influenza virus infection itself significantly alters the composition of the gut microbiota, making it pathologically distinct from other viral diseases such as bacterial pneumonia or COVID-19 (Gu et al., 2020; Lv et al., 2021). This suggests that changes in the gut microbiota can serve as an important biomarker to aid in diagnosis and assessment of disease progression, with great potential for clinical translation. In terms of clinical applications, research on modulating the “gut-lung axis” has gradually become a hot topic, and the related findings may provide new insights and strategies for influenza prevention and control: first, intervening in influenza susceptibility by modulating the gut microbiota (e.g., oral probiotics, prebiotics, or fecal microbiota transplants); second, enhancing the immunogenicity of influenza vaccines and their protective efficacy of influenza vaccines; third, using gut microbiota characteristics for early prediction of influenza infection and personalized diagnostic decision-making to improve precision medicine; and fourth, exploring the clinical efficacy of microbiota interventions in improving the prognosis of influenza in high-risk populations, such as the elderly and patients with chronic diseases. These studies have expanded the understanding of viral infections from a microbiome perspective and improved the clinical relevance of the research. However, the mechanisms of interaction between the gut microbiota and influenza viruses are complex and a systematic research framework is lacking. Currently, there is no bibliometric study dedicated to the “relationship between influenza and gut microbiota” to comprehensively assess the trends, research hotspots, and key scientific issues in this field. To address this gap, this study visualized the global research results in this field from 2011 to 2025 using tools such as VOSviewer and CiteSpace. It systematically summarizes the strengths and weaknesses of existing research in terms of research topic evolution, keyword clustering, high-impact literature, and emerging directions, which provides a theoretical basis and decision-making reference for future basic research and clinical translation.

## 2 Materials and methods

### 2.1 Source database and search strategy

**FIGURE 1:**
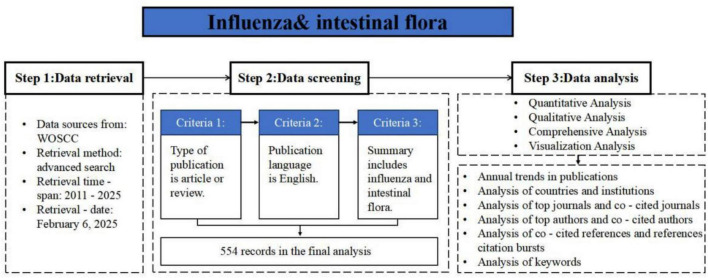
Flow chart of inclusion and exclusion criteria.

Influenza-related articles were retrieved from the Web of Science Core Collection (WoSCC) database. Relevant data up to February 5, 2025 were downloaded to ensure consistent searching of all data. The following key search terms were used: TS = [(“influenza” OR “flu”) AND (“gut microbiota” OR “gut microbiome” OR “intestinal microbiota” OR “intestinal microbiome”)], covering the period from January 1, 2011, to February 5, 2025, was used. A total of 554 literatures were obtained. Based on the inclusion and exclusion criteria, articles were screened by two individuals, resulting in 283 valid publications, and the search framework is shown in Figure 1.

### 2.2 Methods

Bibliometric and visualization analysis was employed to examine the research landscape on the relationship between influenza and intestinal microbiota over the past fifteen years, utilizing specialized software including VOSviewer (v1.6.18), CiteSpace (v6.1), and Scimago Graphica (v1.0.42). VOSviewer excels in large-scale network clustering and enables high-precision thematic categorization through modularity optimization (Van Eck and Waltman, 2010); it facilitates co-occurrence analysis, network visualization, heatmap generation, and cluster analysis of scientific literature, keywords, and authors. Within its visualizations (e.g., co-citation graphs), nodes represent elements (publications, keywords, authors), where node color signifies group association, node diameter corresponds to article count or citation frequency (larger nodes indicating key topics), and inter-node distance reflects relationship strength (closer proximity denotes stronger association). CiteSpace is widely used for visualizing scientific literature analysis, including citation counts, publication volumes, key disciplines/journals, research institutions/collaborations, and authorship analysis; its unique capability is the built-in Kleinberg burst detection algorithm identifying emerging research frontiers (Chen, 2017), and it also performs clustering (e.g., keyword clustering) and burst analysis. In CiteSpace’s co-occurrence maps, node size represents publication count, inter-node links symbolize collaboration, and link thickness indicates collaboration frequency (denser/thicker connections denoting more active co-occurrence or co-citation relationships). The geographic distribution of influenza inflammation studies was visualized using Scimago Graphica alongside VOSviewer; Scimago Graphica, a web-based tool for creating and editing scientific graphs, provides interactive country/region collaboration maps and excels in spatial relationship representation compared to similar tools.

### 2.3 Terminology definitions

To ensure consistency and scientific accuracy in the use of terminology, the following definitions are provided for key concepts frequently mentioned in this paper:

(1) Gut–lung axis

The gut–lung axis refers to the physiological pathway through which the gut and lungs communicate bidirectionally via immune regulation, metabolic products (such as short-chain fatty acids), microbiome composition, and inflammatory mediators. This axis mechanism explains how gut microbiota dysbiosis can influence lung health through the circulatory system or immune pathways, thereby affecting the host’s susceptibility to respiratory viruses (such as influenza viruses) and immune responses. Research on these mechanisms has increasingly become a hot topic in the interdisciplinary field of microbiology and respiratory infections.

(2) Microbiota–lung axis

The microbiota–lung axis is a specific perspective on the “gut-lung axis,” emphasizing the impact of microorganisms themselves (particularly structural changes in the gut microbiota) on the progression of lung diseases. For example, antibiotic-induced gut microbiota dysbiosis can weaken the lung’s immune barrier against influenza viruses, suggesting the potential of microbiome intervention in the management of respiratory diseases.

(3) Translational microbiota intervention (translational microbiota intervention)

This refers to strategies for applying microbiome research findings to clinical practice, including targeted use of probiotics/synbiotics, personalized vaccine optimization based on gut microbiota characteristics, or interventions to improve the immune status of influenza patients through dietary/pharmacological regulation of the gut microbiota. Bibliometrics can assist in identifying current priorities and evidence bases for translational interventions, providing guidance for clinical design.

## 3 Results

### 3.1 Temporal trends in publications

**FIGURE 2:**
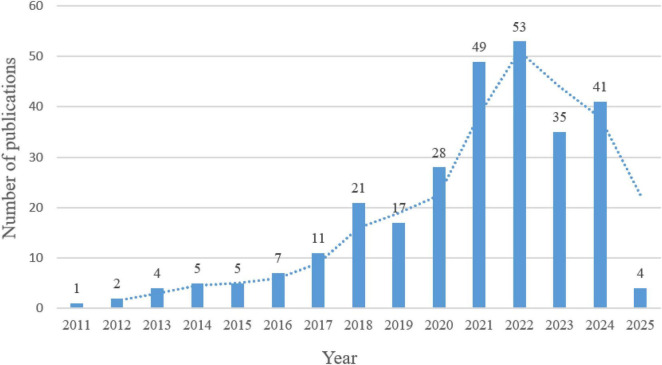
Global publication growth trends, 2011–2025.

The 283 articles were published in 129 journals and were authored by 1945 researchers from 585 institutions in 44 countries/regions. The publication trends for the last 15 years are shown in Figure 2, with incomplete yearly data for 2025; therefore, not shown in Figure 2. Linear regression analysis showed a significant positive correlation between the number of publications and year (β = 3.93, *p* < 0.0001), with a compound annual growth rate of 34.06% during the period 2011–2024.The year 2018 was the key turning point, with an annual increase of 91%, which may reflect the global early warning response to emerging respiratory infectious diseases. The size of research has stabilized at more than four times the pre-pandemic level, despite a small correction after 2022. publications increased significantly after 2018, likely due to the 2019 novel coronavirus pandemic. Publications steadily increased, reaching a peak of 53 publications from 2022, followed by a slight decline, which may explain the end of the global COVID-19 pandemic. These findings suggest that influenza gut flora has become increasingly topical in recent years, especially in influenza research.

### 3.2 Analysis of countries/regions and institutions

**FIGURE 3:**
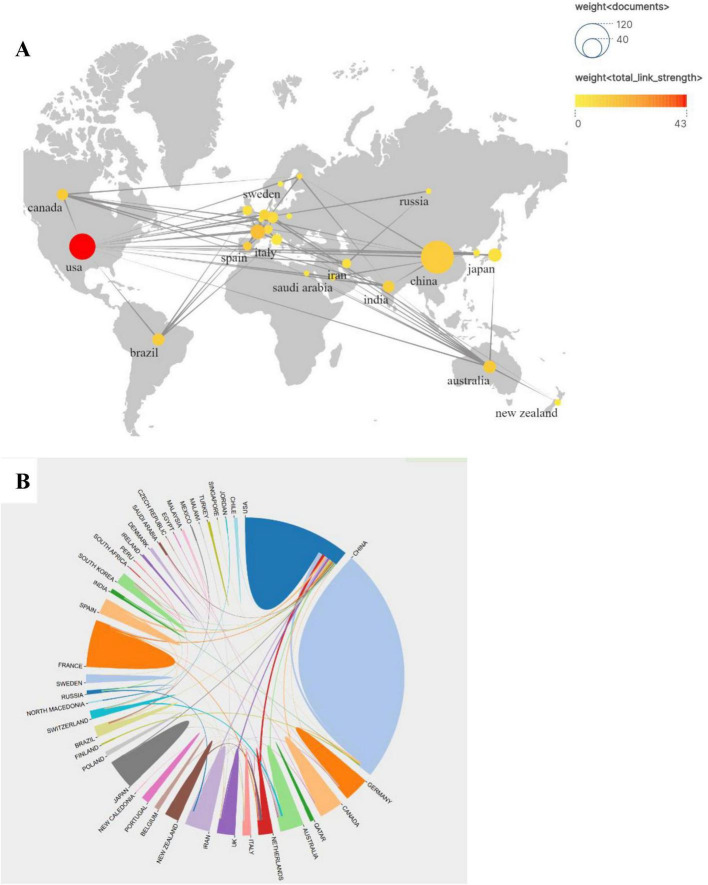
**(A)** Map based on publications and partnerships showing the geographical distribution of publications by country/region. **(B)** Map showing publications co-authored by Country/Region.

Nearly 50 countries or regions are involved in influenza and gut microbiology research. Figure 3A shows the dissemination and the number of publications and collaborations in these countries/regions. The size and color shades of the different circles represent the number of publications in each country and the strength of cooperation between countries, respectively. The larger circle represents more publications, and the darker color represents the stronger cooperation between this country and other countries, among which the largest number of publications is from China (97), and the largest strength of cooperation is from the U.S., with the Cooperation Strength Index (CSI) of 0.72 (calculated by the number of coauthored papers/total number of papers) (calculated by the number of co-authored papers/total papers). The publication outputs of the top 10 countries/regions and institutions are listed in Table 1. Among the top 10 countries, 3 countries, Japan, China, and India, are Asian countries with 127 (45%) of their total publications, mainly focusing on the study of influenza virus-intestinal flora interactions mechanisms. Collaboration between the United States and European countries accounted for 47% of the total output, and Germany and France formed a joint research network through the European Union Horizon program, focusing on exploring the clinical translation of probiotic intervention in influenza.

**TABLE 1:** Top 10 countries/territories and institutions in terms of number of articles published.

Rank	Country	Records	Organization	Records
1	China	97	Zhejiang University	10
2	USA	65	University of Lille	9
3	France	20	Capital Medical University	8
4	Japan	17	Institute Pasteur	6
5	Australia	15	University of Toronto	6
6	Brazil	14	Beijing Institute of Chinese Medicine	5
7	India	13	Beijing University of Chinese Medicine	5
8	Canada	12	Shanghai University of Traditional Chinese Medicine	5
9	Germany	12	University of Guelph	5
10	Italy	11	University of Paris-Saclay	5

Furthermore, numerous international collaborative networks have been established across multiple countries and regions. As illustrated in Figure 3B, both the quantity of published research articles and the intensity of cross-border academic partnerships exhibit distinct distribution patterns. Notably, the United States demonstrates the highest publication output while engaging in multi-national research alliances that notably include China as a key partner. Additionally, China, the United States, and France have developed extensive collaborative engagements with global counterparts through multidisciplinary research initiatives.

### 3.3 Analysis of top ranked journals and co-cited journals

**FIGURE 4:**
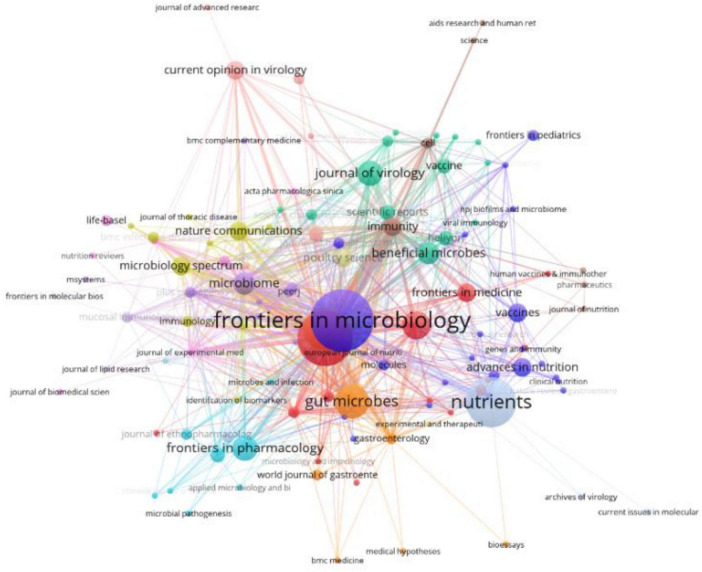
Visualization of journals on the relationship between influenza and gut microbiota.

A total of 129 journals published research related to influenza and gut microbiota. Frontiers in Microbiology published the most number of articles (20), followed by Frontiers in Immunology (17) and then Nutrients (15). A total of 283 publications were referenced across 2,887 distinct journals, with distribution patterns visualized in Figure 4. Table 2 further details the top 10 most-cited journals, revealing *Nature* as the foremost source (605 citations), followed by *Frontiers in Immunology* (585 citations) and *Science* (582 citations). *Cell* also featured prominently within this cohort, accumulating 477 citations. Notably, six journals (in 2025) had IF scores of 10 or more, with Nature topping the list at 42.7. The high co-citation frequencies of Nature and Science illustrate that the field of influenza and intestinal flora research is built on landmark, interdisciplinary breakthroughs in basic biology. These breakthroughs established gut flora as a key factor in influenza outcomes. Meanwhile, the high co-citation frequency of Frontiers in Immunology suggests that immunological mechanisms are at the core of the field and are the most active area of research, with a large number of studies devoted to analyzing the specific pathways by which flora and their products modulate host immunity against influenza. The high frequency of all three together in the co-citation network strongly suggests that the field is a cutting-edge hotspot, a feature accurately captured by the bibliometric results.

**TABLE 2:** Top 10 journals and co-cited journals in terms of literature volume.

Rank	Journals	Counts	IF	Co-cited journals	Co-cited counts	IF
1	Frontiers in Microbiology	20	4.259	Nature	605	42.778
2	Frontiers in Immunology	17	3.517	Frontiers in Immunology	585	3.517
3	Nutrients	15	4.546	Science	582	41.845
4	Frontiers in Cellular and Infection Microbiology	12	3.303	PLoS One	547	3.752
5	Gut microbes	9	9.433	Immunity	506	31.492
6	Microorganisms	7	4.157	Proceedings of the National Academy of Sciences (PNAS)	490	12.78
7	Frontiers in Pharmacology	6	3.845	Cell	477	41.582
8	Journal of Virology	6	4.456	Frontiers in Microbiology	336	4.259
9	Beneficial Microbes	5	3.942	Journal of Immunology	321	5.02
10	Microbiome	5	14.65	Cell Host & Microbe	295	21.02

**FIGURE 5:**
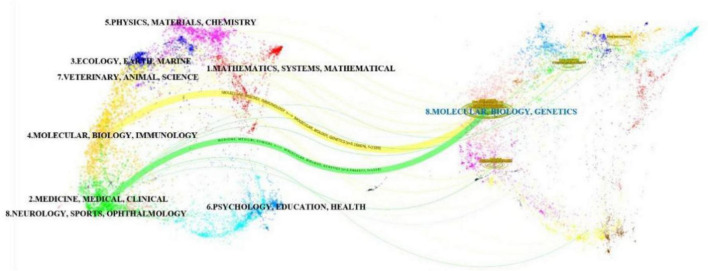
Overlay of journals related to influenza and intestinal flora research.

The journal overlay visualization (Figure 5) employs subject-based clustering to construct dual bibliometric matrices: a journal citation network (left) and a journal cited network (right). Inter-map co-citation linkages reveal disciplinary knowledge transfer dynamics. Elliptical datapoints represent journal entities, with the horizontal axis quantifying publication volume. Notably, the Molecular/Biology/Genetics (MBG) domain exhibits maximal density in both authorship and article output. Two primary clusters emerge in the citing map: Medicine/Medical/Clinical (MMC) and Molecular/Biology/Immunology (MBI). Conversely, the cited map demonstrates disciplinary convergence toward a unified MBG cluster, signifying evolving research paradigm integration and foundational science dominance in knowledge dissemination. Through this visualization analysis, the research dynamics, knowledge flow, and inter-disciplinary interactions in various disciplinary fields can be more clearly understood, providing valuable references for future research directions and collaborations.

### 3.4 Analysis of top authors and co-cited authors

**FIGURE 6:**
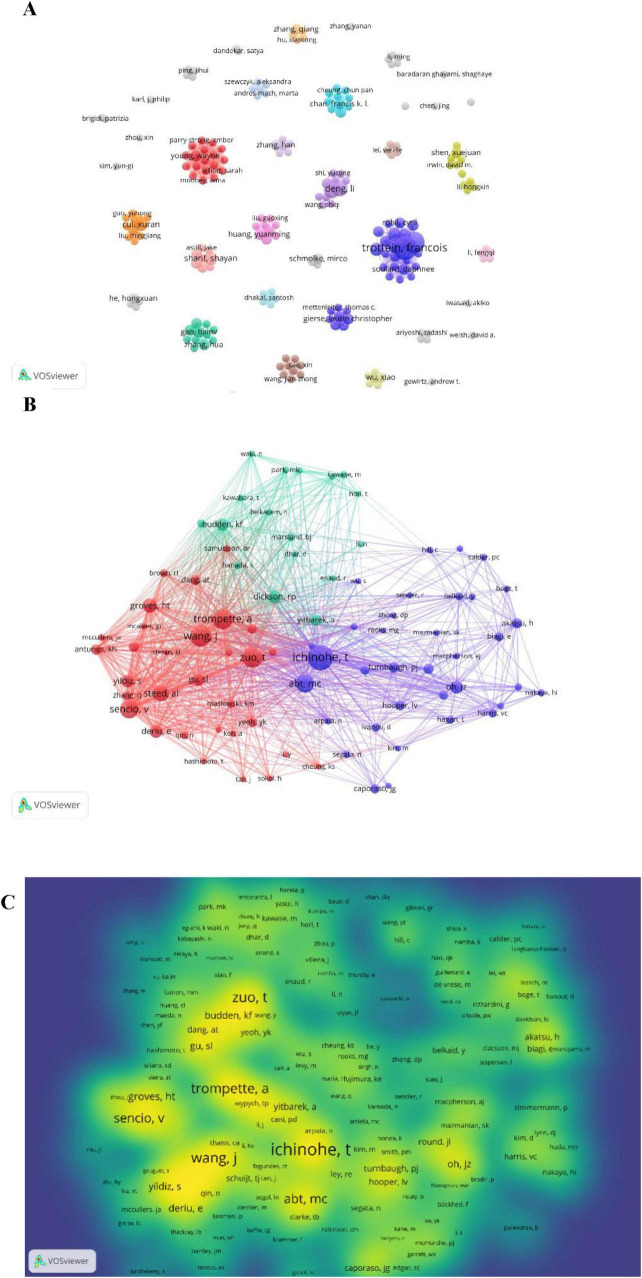
**(A)** Collaboration between authors. **(B)** Co-cited authors. **(C)** Density map of co-cited authors.

Over the past 15 years, 1,945 researchers have investigated the influenza-gut microbiota relationship. Their collaborative networks (Figure 6A) reveal globally dispersed but regionally concentrated partnerships, with most scholars engaging in limited collaborations. Table 3 details the top 10 most prolific authors ranked by publication volume. Prof. Francois Trottein is ranked first with 9 articles. Francois Trottein’s research focuses on the interaction of influenza virus infections with the intestinal flora and its effect on host immunity. His research found that influenza virus not only affects the respiratory tract, but also leads to intestinal dysbiosis, which is characterized by a decrease in the number of beneficial bacteria and an increase in the number of harmful bacteria, which in turn affects the intestinal barrier function and triggers a systemic inflammatory response. In addition, intestinal flora and their metabolites (e.g., bile acids, short-chain fatty acids, etc.) are able to modulate the immune response of the host and enhance the resistance to influenza virus. Based on these findings, modulation of gut flora (e.g., through probiotic or dietary

**TABLE 3:** Top 10 prolific and co-cited authors and their citation frequency.

Rank	Author	Count	Citations	Average citations	Co-cited author	Co-cited counts
1	Francois Trottein	9	684	76.00	T. Ichinohe	126
2	Valentin Sencio	8	601	75.13	J. Wang	99
3	Li Deng	5	89	17.80	T. Zuo	95
4	Isabelle Wolowczuk	5	340	68.00	A. Trompette	94
5	Huachong Xu	5	75	15.00	V. Sencio	84
6	Xuran Cui	4	14	3.50	M.C. Abt	83
7	Corinne Grangette	4	117	29.25	A.L. Steed	70
8	Severine Heumel	4	117	29.25	H.T. Groves	60
9	Qingquan Liu	4	14	3.50	S. Yildiz	53
10	Marina Gomes Machado	4	287	71.75	E. Deriu	52

interventions) may become a new adjunctive therapeutic strategy to help reduce influenza symptoms and accelerate recovery. These studies provide new ideas and directions for the treatment of influenza.

Table 3 details the top 10 most co-cited authors and their citation frequencies. The rankings in order are T. Ichinohe (126 citations), J. Wang (99 citations), T. Zuo (95 citations), A. Trompette (94 citations), and V. Sencio (84 citations). Figure 6 shows the co-citation relationship between authors. Figure 6C shows a density plot of the influence of co-cited authors.

### 3.5 Analysis of co-cited references and reference citation bursts

**FIGURE 7:**
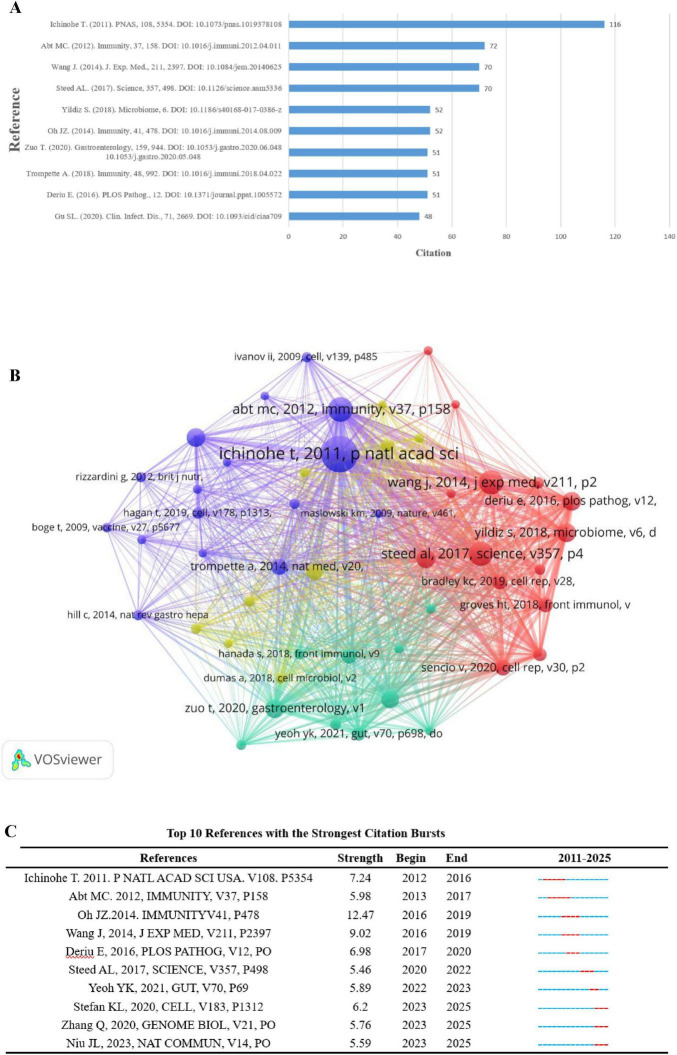
**(A)** Top 10 co-cited literature **(B)**. Network of co-cited publications cited at least 20 times **(C)**. Top 10 articles by intensity of cited outbreaks.

A co-cited reference is defined as an article that reveals the importance of the article in a particular field when it is cited by two or more publications. Among them, the research paper “Microbiota regulates immune defense against respiratory tract influenza A virus infection” by Akeshi Ichinohe et al. published in Proc Nat Acad Sci U S A in 2011. A virus infection published in Proc Nat Acad Sci U S A in 2011 ranked first with 116 citations (Figure 7A). It was found that the composition of the symbiotic microbial community has a significant impact on the production of virus-specific CD4 and CD8 T cells and antibody responses. By using different antibiotic treatments, the investigators found that neomycin-sensitive bacteria were associated with the production of an effective immune response in the lungs, and the findings reveal the importance of the commensal microbiota in modulating respiratory mucosal immunity through appropriate activation of inflammatory vesicles. As shown in Figure 7A. Co-cited articles (more than 20 citations) and network diagrams, as shown in Figure 7B.

Literature burst analysis is used to detect high-frequency and fast-growing bursts of literature by examining the temporal distribution of literature, and then analyzing the cutting-edge areas and trends of a discipline. Citation burst detection uses a sensitivity setting of γ = 1.0 (Kleinberg, 2002) to ensure that significantly growing but non-noisy literature is identified. Figure 7C shows the 10 highest citation bursts sorted by burst intensity. The first citation burst occurred in 2012 and the most recent in 2023. The longest duration was 5 years. M. C. Abt’s 2012 article in the journal Immunity, “Commensal bacteria calibrate the activation threshold of innate antiviral immunity,” focuses on how commensal bacteria regulate the activation threshold of innate antiviral immunity. The article states that symbiotic bacteria modulate the host’s immune response to viral infection by maintaining a homeostasis. Specifically, the presence of commensal bacteria influences the host cell’s recognition of and response to viruses, thereby affecting the level of activation of innate immunity. This study reveals the interactions between the gut microbiota and the host immune system, emphasizing the important role of the microbiota in modulating the immune response.

### 3.6 Keyword analysis

**FIGURE 8:**
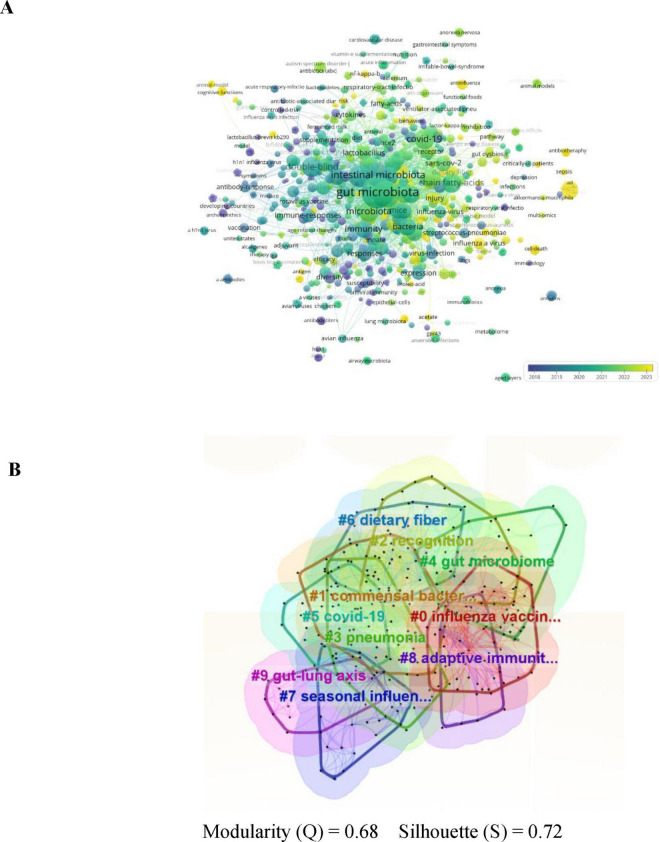
**(A)** Keyword co-occurrence network. **(B)** Keyword co-occurrence clustering map.

The role of keywords is crucial to accurately summarize the core content and themes of the study. Keyword analysis provides a comprehensive overview of influenza and gut microbiota research, prevalent themes, and emerging trends for future research. Figure 8A shows the keyword co-occurrence network diagram, which visually represents the frequency of keywords and visually reveals the focus of the study. The circle size of the keyword co-occurrence network diagram indicates the frequency of keyword occurrence, the higher the frequency, the larger the circle; the color shades indicate the changes of different keywords at different times. The 10 keywords with the highest frequency of occurrence are shown in Table 4, and it is worth noting that the top three are “gut microbiota” (*n* = 139, 9.6%), “influenza” (*n* = 88, 6.0%) and “Intestinal microbiota” (*n* = 88, 6.0%), respectively.), and “Intestinal microbiota” (*n* = 63, 4.3%), indicating that gut microbiota, influenza, and Intestinal microbiota are highly relevant topics for this study. In this study, after clustering developed the clustering map all keywords, the result of keyword clustering in Figure 8B shows that modularity Q = 0.4478, where when Q > 0.3, indicates a significant structure, and contour coefficient S = 0.7311, where when s > 0.7, indicates strong cohesion, which suggests that the topics are categorized reasonably and with a high degree of internal consistency. The clustered keywords showed that commensal bacteria, gut microbiome and COVID-19 formed tight clusters, which provided a strong support for exploring the mechanism of influenza-fungus interactions.

**TABLE 4:** Top 10 most frequent keywords.

Rank	Keyword	Counts
1	Gut microbiota	139
2	Influenza	88
3	Intestinal microbiota	63
4	Probiotics	53
5	Microbiota	44
6	Double-blind	42
7	COVID-19	41
8	Infection	39
9	Chain fatty acids	38
10	Inflammation	37

## 4 Discussion

This study employed five analytical tools to visualize bibliometric data from 283 publications, systematically evaluating research status and emerging trends in influenza-modulated gut flora through quantitative, qualitative, and integrative methodologies. Significantly, it constitutes the first bibliometric analysis in this field.

### 4.1 Trends in influenza-related research

Influenza research has made important progress in recent years, especially in deepening the understanding of the disease phenotype and classification. However, there is a lack of international consensus on the relationship between influenza and intestinal flora, and there are still research gaps in pathogenesis, treatment and prognosis. Currently, there are no bibliometric analyses exploring the relevant associations between influenza and gut flora. This study aims to summarize the research trends in this field and provide a reference for related studies. The results showed that the annual publication volume of influenza and intestinal flora research has increased significantly since 2019, reflecting the increasing research fervor in this field. Currently, about 50 countries have published relevant papers, with China having the largest research output (97), the United States the next largest (65), and France (20) and Japan (17) having less but influential studies. For example, Francois Trottein from France and T. Ichinohe from Japan have made outstanding contributions in this field.

Institutional collaborations, such as Zhejiang University and Capital Medical University in China, form a domestic research cluster, while the University of Lille and Institut Pasteur in France dominate in Europe. Overall, more than 70% of the global relevant research is done by the top five countries, showing the role of leading institutions in driving academic impact.

In the future, research in this field can further strengthen international cooperation, improve research quality and promote the output of high-level results to deepen the understanding of the relationship between influenza and intestinal flora.

### 4.2 Status and quality of authors, journals and research

In recent years, Chinese scholars have made outstanding contributions to the field of influenza and intestinal flora research, and have led the way in terms of the number of papers published and their impact. However, international collaborations are relatively decentralized, and academic exchanges between different countries still need to be strengthened to promote more in-depth research. Most of the relevant papers were published in microbiology and immunology journals, among which Frontiers in Immunology is a high-impact journal preferred by researchers. In terms of co-citation analysis, Nature has a significant impact in the field, publishing papers that lay theoretical foundations or present key findings. Since 2018, research in this field has continued to grow, with more than 20 papers published each year, and more explosive growth after 2019, emphasizing its research value. The United States collaborates closely with countries such as China, Canada, and Japan to advance the field. In terms of scholars’ contributions, Francois Trottein (9 papers) is a prolific author, and T. Ichinohe (126 citations) and J. Wang (99 citations) are widely recognized for their research results, and may be the founding fathers of the field. For example, T. Ichinohe focuses on viral-host immune interactions, while Francois Trottein and Valentin Sencio focus on the relationship between gut microbes and the immune system. Frontiers in Microbiology has the largest number of publications (20). Among the highly cited journals, Nature (605 co-citations) and Science (582 co-citations) are the most influential, which shows that the research quality in this field is high, and the cutting-edge results are mainly focused on microbiome-host immune interactions, infection immunity and metabolic regulation. In the future, this field should strengthen international cooperation, promote high-level research, and deepen the understanding of the relationship between influenza and intestinal flora.

### 4.3 Research hot spots and frontiers

With the development of microbiome technology, the interaction between influenza and gut microbiota (GM) has become a research hot spot. Studies have shown that gut microbiota can regulate the host immune response to influenza A virus (IAV) through the “gut-lung axis,” and its metabolites (e.g., short-chain fatty acids, secondary bile acids) may be biomarkers and therapeutic targets for influenza infection (Stefan et al., 2020; Zhang et al., 2022). Gu et al. (2020) found significant differences in the pattern of intestinal dysbiosis between COVID-19 and H1N1 influenza patients, suggesting that bacterial flora characteristics can be used to differentiate between types of viral infections. In addition, it has been shown that the gut fungal and viral groups may also influence influenza immunity, e.g., fungal abundance is negatively correlated with host antiviral gene expression (Lv et al., 2021).

In terms of interventions, probiotics, postbiotics, and flora transplantation have demonstrated the potential to modulate Th17/Treg homeostasis, enhance the mucosal barrier, and mitigate lung injury in animal models (Kawase et al., 2010; Hu et al., 2021). However, the dynamics of gut flora-host immune interactions, metabolite-mediated signaling pathways and their clinical applications still need to be thoroughly investigated. In this section, we will discuss the latest advances and challenges in this field, focusing on immunopathological mechanisms, flora intervention strategies and future directions.

#### 4.3.1 Immunopathogenesis

Influenza A virus (IAV), SARS coronavirus (SARS-CoV), and SARS-CoV-2 are the major pathogens responsible for severe pneumonia (Figueiredo, 2009), posing a major threat to public health and the global economy (Nair et al., 2010). Influenza viruses are the most common respiratory pathogens, and the World Health Organization estimates that influenza epidemics result in approximately 1 billion infections each year, of which 3–5 million are severe cases and 300,000–500,000 deaths (Singhal, 2020). The efficacy of anti-influenza drugs relies on early diagnosis and accurate viral typing, while the effectiveness of vaccines is limited by the high variability of the virus (Krammer et al., 2018; Yen, 2016). Therefore, it is crucial to develop alternative medical interventions and economically viable treatment strategies.

Following IAV infection, pattern recognition receptors (PRRs) are activated, triggering excessive release of inflammatory factors and chemokines (Dewe et al., 2013), which may lead to airway and alveolar epithelial damage, and even trigger acute respiratory distress syndrome (ARDS) and multi-organ dysfunction (Klomp et al., 2021). Meanwhile, ischemia and hypoxia induced by lung infection can damage the intestinal barrier and lead to intestinal flora disorders, which in turn affects lung-intestinal mucosal immunity and aggravates lung injury (Gu et al., 2019). A growing body of research supports the role of the “gut-lung axis” in influenza infection (Herold et al., 2015). For example, IAV infection leads to increased intestinal permeability, decreased cuprocytes, decreased tight junction protein ZO-1, and triggers an imbalance in intestinal flora that exacerbates lung injury in mice (Dang and Marsland, 2019; de Oliveira et al., 2021). In addition, modulation of Th17/Treg balance in gut-associated lymphoid tissues helps to ameliorate lung inflammation (Chen et al., 2017). These studies shed light on the mechanisms of IAV-induced lung-intestinal immune injury and have propelled the field into a research hotspot.

#### 4.3.2 Gut flora regulation of influenza A virus infection

The gastrointestinal tract, recognized as the body’s paramount immune organ, relies on dynamic gut microbiota (GM) modulation to mitigate a spectrum of inflammation-associated pathologies—spanning inflammatory bowel disease, malignancies, neurodegenerative disorders like Alzheimer’s disease, and mood dysregulation including depression (Lavelle and Sokol, 2020; Fung et al., 2017; Zhou et al., 2021). Clinically, a meta-analysis of 726 patient cases substantiates that probiotic supplementation (e.g., *Lactobacillus*, *Bifidobacterium*, *Lactococcus* strains) significantly attenuates susceptibility to respiratory viral pathogens (Shi et al., 2021). Mechanistically, probiotics, prebiotics, and phytotherapeutics orchestrate innate/adaptive immune recalibration, potentiating pathogen eradication while suppressing cytokine-driven tissue injury. Empirical evidence demonstrates that oral administration of lyophilized *Lactobacillus plantarum* GG and TMC0356 curtails pulmonary viral titers in influenza A (PR8 strain)-challenged murine models (Kawase et al., 2010); this antiviral phenotype extends to *L. plantarum* 06CC2, DK119, and *L. paracasei* via synergistic induction of antiviral gene transcription (*Mx1*, *Oas1a*, etc.) (Nakayama et al., 2014; Takeda et al., 2011; Park et al., 2013). Furthermore, select probiotic consortia enhance dendritic cell chemotaxis, amplify T-lymphocyte effector functions, and elevate secretory profiles of interferon-α/γ and interleukin-12—collectively fortifying mucosal antiviral immunity (Xing et al., 2022; Belkacem et al., 2017; Kobayashi et al., 2011).

**FIGURE 9:**
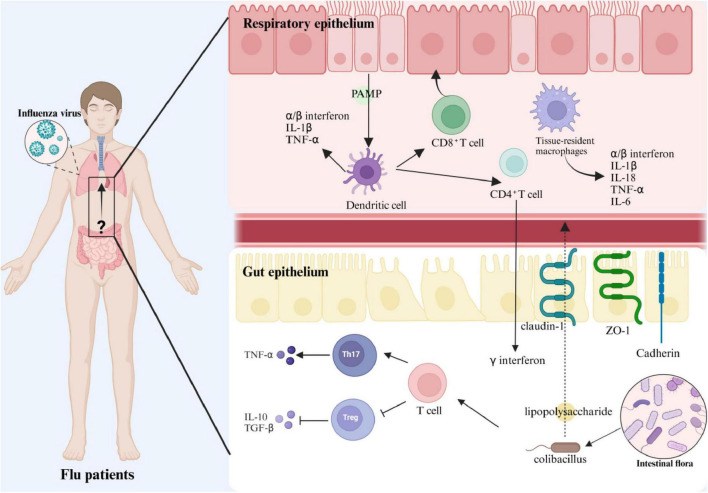
Schematic representation of the immune mechanism of intestinal flora regulating influenza A virus infection.

During the post-viral resolution phase, probiotics demonstrate dual therapeutic capacities by mitigating inflammatory pathology and augmenting tissue regeneration. Empirical studies reveal *Lactobacillus rhamnosus* M21 significantly attenuates pulmonary inflammation in influenza-infected murine models (Song et al., 2016), while *Akkermansia muciniphila* orchestrates pro-/anti-inflammatory cytokine equilibrium and *Bacillus breve* CCFM1026 elevates intestinal butyrate production to suppress distal lung immunopathology (Hu et al., 2021; Lu et al., 2021). Critically, select probiotic strains exhibit temporal duality: enhancing viral clearance during early infection while suppressing cytokine storms in later phases, thereby establishing bidirectional immunomodulatory paradigms. Influenza virus infection also damages the intestinal barrier, shortening and shedding intestinal villi and decreasing the expression of tight junction proteins (claudin-1, ZO-1), which increases intestinal permeability and exacerbates the inflammatory response (Guo et al., 2024). As shown in Figure 9, the regulatory effects of probiotics may provide new strategies to alleviate infection-related inflammation and protect the intestinal barrier.

### 4.4 Future research trends

The gastrointestinal tract constitutes the body’s principal immune organ, with gut microbiota (GM) serving as master regulators in the pathogenesis of immune-mediated diseases. Contemporary research on the “gut-lung axis” has illuminated GM’s critical involvement in respiratory infections, particularly influenza A virus (IAV). Yet viral and fungal components—dubbed “microbial dark matter”—have been historically marginalized due to low biomass and technical constraints in conserved viral genome identification (Wang, 2020).

Advancements in metagenomic sequencing now facilitate comprehensive profiling of enteric viromes and mycobiomes. Defining IAV-induced alterations in these communities could unravel enterovirus-modulated gut-pulmonary crosstalk during antiviral defense. Fundamentally, shared immunological pathways exist: IAV recognition through TLR3/7/8 and RIG-I/MDA5 sensors, NLRP3 inflammasome activation, and subsequent adaptive responses—including germinal center B-cell maturation, CD4^+^ T follicular helper cell polarization, and CD8^+^ cytotoxic T-lymphocyte recruitment—are metabolically tuned by GM-derived short-chain fatty acids and secondary bile acids.

Despite anatomical compartmentalization, bidirectional gut-lung communication occurs via immune cell trafficking and microbial metabolite dissemination. Current literature predominantly documents correlative links between GM dysbiosis and pulmonary immune markers (e.g., IFN-λ, IL-33) during IAV infection. Critical knowledge gaps persist regarding causal mechanisms, including bacteriophage-mediated horizontal gene transfer, β-glucan-induced trained immunity, and neuro-immune signaling—necessitating gnotobiotic models and spatiotemporal multi-omics integration for mechanistic dissection.

### 4.5 Strengths and limitations

The significant strength of this study is that it has systematically sorted out the knowledge structure and evolution path of the research field of “influenza-intestinal flora” from the perspective of scientific literature. This study is based on traditional bibliometric tools such as VOSviewer and CiteSpace, and from the perspectives of keyword clustering and co-occurrence analysis, this kind of method has good applicability in analyzing the development trend of disciplines, the distribution of research hotspots and the cooperation network at the macro level due to the standardization of the parameter settings, the clear structure of the map, and the ease of interpreting the results, and is especially suitable for large-scale data analysis of cross-disciplines and cross-institutions. It is especially suitable for interdisciplinary and inter-institutional large-scale data analysis.

However, we also realize that there is still room for improvement in the traditional methods in terms of revealing the time sensitivity of thematic evolution and the structural explanatory power of complex networks. In recent years (2023–2024), related studies have proposed a variety of hybrid modeling approaches based on the fusion of semantic embedding, machine learning and Altmetric metrics. For example, Zhao et al. significantly improved the explanatory depth of the literature co-citation network and the accuracy of trend identification by introducing structural feature recognition and machine learning clustering (Zhao et al., 2024); another study incorporated Altmetric attention data into the analysis framework, which enhances the ability to capture emerging research frontiers, especially for rapidly evolving cross-disciplinary fields (Zhao et al., 2022).

If future research can integrate semantic analysis, natural language processing (NLP) and artificial intelligence algorithms on the basis of traditional visual analytics, it is expected to build a more dynamic and predictive research map, and realize the leap from static knowledge presentation to intelligent topic evolution analysis. This will not only help identify potential research breakthroughs in a timely manner, but also provide a clearer pathway to support the translation between basic research and clinical applications, further enhancing the strategic value and practical impact of the “Influenza—Intestinal Flora” field.

## 5 Conclusion

This study employed bibliometric methods to systematically analyze the global research landscape on “influenza and gut microbiota” from 2011 to 2025, including publication trends, collaborative networks, and emerging research hotspots. The findings indicate a sustained rise in scholarly interest, particularly focused on the mechanisms underlying the interaction between influenza virus infection and the gut microbial ecosystem. Notably, multiple countries and research institutions have established stable collaborative frameworks that are accelerating the integration of basic research with clinical applications.

In recent years, the focus of research has gradually shifted toward investigating how influenza impacts gut barrier function, modulates intestinal immune responses, and influences disease progression through the microbiota–immune axis. High-frequency keyword clustering and burst term analysis reveal that concepts such as the “gut–lung axis” and “probiotics in antiviral immunity” have emerged as central themes, offering clear directions for future mechanistic investigations. Importantly, this study provides clinically relevant insights and practical guidance for researchers. For instance, emerging themes such as “probiotic-assisted enhancement of influenza vaccine immunogenicity,” “microbiota-targeted interventions to reduce infection risk,” and “gut microbial signatures predicting influenza severity” represent actionable avenues for translational research. A representative example is the meta-analysis by Zimmermann and Curtis (2019), which demonstrated that probiotic supplementation can enhance post-vaccination antibody responses, highlighting the potential of microecological interventions as adjunct strategies in influenza prevention.

Additionally, the co-authorship and institutional collaboration maps constructed in this study can assist research administrators and funding bodies in identifying high-impact teams and prolific institutions, facilitating strategic resource allocation, grant planning, and multicenter collaborative efforts. Looking ahead, integrating microbiome omics data with artificial intelligence algorithms offers promising prospects for developing individualized influenza risk prediction models and microbiota-driven nutritional interventions. Such approaches will support the construction of a precision intervention framework linking microecology, immunity, and infection, thereby expanding the clinical and public health relevance of this research domain.

In conclusion, this study not only delineates the structural evolution and thematic trajectory of the “influenza–gut microbiota” field but also provides theoretical insights and methodological references for advancing cross-disciplinary collaboration, optimizing research design, and accelerating the clinical translation of findings. It holds considerable value for both public health strategy development and future clinical research planning.

## Data Availability

The original contributions presented in the study are included in the article/supplementary material, further inquiries can be directed to the corresponding author.
